# The missing link in parasite manipulation of host behaviour

**DOI:** 10.1186/s13071-018-2805-9

**Published:** 2018-04-03

**Authors:** Ryan Herbison, Clement Lagrue, Robert Poulin

**Affiliations:** Department of Zoology, University of Otago, P.O. Box 56, Dunedin, 9054 New Zealand

**Keywords:** Parasite, Behaviour, Manipulation, Mechanism, Manipulation factor

## Abstract

The observation that certain species of parasite my adaptively manipulate its host behaviour is a fascinating phenomenon. As a result, the recently established field of ‘host manipulation’ has seen rapid expansion over the past few decades with public and scientific interest steadily increasing. However, progress appears to falter when researchers ask how parasites manipulate behaviour, rather than why. A vast majority of the published literature investigating the mechanistic basis underlying behavioural manipulation fails to connect the establishment of the parasite with the reported physiological changes in its host. This has left researchers unable to empirically distinguish/identify adaptive physiological changes enforced by the parasites from pathological side effects of infection, resulting in scientists relying on narratives to explain results, rather than empirical evidence. By contrasting correlative mechanistic evidence for host manipulation against rare cases of causative evidence and drawing from the advanced understanding of physiological systems from other disciplines it is clear we are often skipping over a crucial step in host-manipulation: the production, potential storage, and release of molecules (manipulation factors) that must create the observed physiological changes in hosts if they are adaptive. Identifying these manipulation factors, *via* associating gene expression shifts in the parasite with behavioural changes in the host and following their effects will provide researchers with a bottom-up approach to unraveling the mechanisms of behavioural manipulation and by extension behaviour itself.

## Letter to the Editor

Behaviour, as a physical concept, presents an incredibly complex challenge to researchers looking to understand its inner workings. Parasites capable of behavioural manipulation provide a direct window into the molecular basis of behaviour by altering physiological systems in their hosts to generate behaviours that align with their life-cycle [1, 2]. The potential of manipulative parasites to increase our fundamental understanding of behaviour is now beginning to dawn on the wider scientific community [1, 3, 4] as is the fact that some of the world’s deadliest diseases (such as malaria) rely on behavioural manipulation to complete their life-cycle [5]. Consequently, studies investigating the molecular mechanisms behind parasite manipulation are slowly increasing in frequency [6, 7]. However, the molecular evidence for adaptive behavioural manipulation (behavioural change in the host that directly benefits the parasite) in a majority of host-parasite associations is missing crucial pieces of evidence. If we define establishment of a parasite in its host (or physical contact with the host by the parasite) as **Event A** and the resulting physiological change in the host as **Event B,** we can now consider the evidence that connects **Event A** to **Event B** in the three cases of host manipulation that follow**:**

- Case 1: A rat contracts *Toxoplasma gondii via* contact with cat faeces (**Event A**). Following this, testicular testosterone production increases in the rat leading to hypomethylation of the amygdala (**Event B**). This is suggested to cause the loss of aversion to cat urine in the infected rats [8, 9].
- Case 2: A mouse contracts *Leishmania amazonensis via* a sand fly bite (**Event A**). Two to four months post-establishment, several key cytokines levels are altered in the mouse’s pre-frontal cortex (**Event B**), potentially resulting in a set of observed anxiety behaviours in infected mice [10].
- Case 3: The parasitoid wasp *Cotesia congregata* injects an egg into the caterpillar *Manduca sexta* (**Event A**). During egression of the larvae from the caterpillar, removal of octopamine from the hemolymph of the caterpillar is significantly reduced (**Event B**). This is thought to suppress the caterpillar’s feeding, thus enabling the larvae to egress unimpeded [11].

**Fig. 1:**
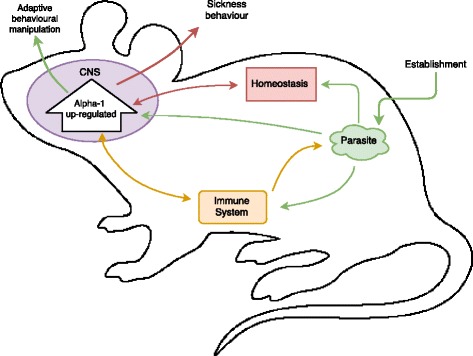
Hypothetical host-parasite system where parasite establishment in the host coincides with Alpha-1 (neurochemical/hormone) upregulation in the central nervous system (CNS) of the host. Arrows demonstrate multiple potential explanations for alpha-1 upregulation. *Green arrows*: Parasite may directly impact Alpha-1 regulation resulting in adaptive behavioural manipulation, but it may also trigger homeostatic and/or immunological mechanisms of the host. Alpha-1 upregulation could also be a by-product of the real mechanism for manipulation or an immune evasion strategy by the parasite. *Orange arrows*: Parasite establishment may stimulate immunological defense mechanisms which require the upregulation of Alpha-1, or conversely increased Alpha-1 presence may be a side effect of immunlogical activation from parasite establishment. *Red arrows*: Parasite establishment may trigger mechanisms involving alpha-1 designed to maintain homeostasis. By extension, Alpha-1 could be primarily involved in inducing sickness behaviours designed to return the host to homeostasis

As with a majority of host manipulation research, the three cases presented above cannot categorically connect parasite establishment (**Event A**) with the physiological/molecular changes seen in infected hosts (**Event B**). Instead, one must correlate parasite presence with the observed physiological changes that coincide temporally with one or another stage of infection. Consequently, this makes it extremely difficult to distinguish adaptive physiological changes induced by the parasites from other outcomes of infection. The reported physiological changes in the hosts from the cases given above could have multiple alternative explanations involving, but not restricted to, immunological or homeostatic mechanisms (see Fig. 1 for illustration). Empirical research needs to generate evidence that allows us to rule out these alternative explanations in order to confirm adaptive behavioural manipulation by the parasite. Failing this, convincing narratives pushing for adaptive manipulation will continue to play an overly important role in this research area. For example, in a review of *Toxoplasma*’s host manipulation mechanisms by Vyas [9] we see the multiple potential pathways framed as ‘narratives’ by the author.

In what follows, we attempt to further clarify the thought process needed to generate definitive evidence for adaptive behavioural manipulation. By using an analogy from a different discipline and the jewel wasp-cockroach host-parasitoid system, and comparing them to the cases described above, we hope to clearly point out the gaps in our present knowledge. Furthermore, we suggest a step-by-step process that parasites should logically follow to adaptively manipulate behaviour and propose a potential avenue for researchers to begin generating evidence for the currently overlooked steps. Note that we are in no way dismissing earlier empirical studies or undermining their importance. Indeed, correlative evidence is a crucial stepping-stone toward uncovering the underlying causal mechanisms, and earlier results may point towards the true mechanisms of host manipulation. However, there is a dire need to push the field of host manipulation study to the next step and the search for the actual causative mechanisms.

### Tobacco and Jewel wasps

In order to demonstrate the correlative nature of many earlier host manipulation studies, allow for an analogy: consider the addiction induced by smoking tobacco as a form of behavioural manipulation, and compare this against the cases presented above. Smoking tobacco (**Event A**) causes the nicotine stored in the plant leaves to be released and inhaled into the lungs where it rapidly travels to the brain. Once there, it saturates receptors normally reserved for binding acetylcholine, culminating in the excessive release of dopamine in the brain (**Event B**). Dopamine signals pleasure and is crucial in reinforcing behaviours. Constant, large releases of this neurochemical quickly result in addiction to nicotine and thus smoking [12]. Albeit simplified, this system of behavioural modification has clear causative evidence, specifically the release of nicotine, connecting **Event A** and **B**. Consider a correlative explanation for tobacco addiction, similar to the three host manipulation cases presented above, in which we would only know that smoking tobacco results in higher dopamine levels in the brain, but have no idea of nicotine’s existence. Nicotine is the *causative factor* (i.e. the substance released by smoking tobacco) directly triggering the cascade of processes leading to addiction. Identification of nicotine and its role marked the transition from correlation to causation in research on tobacco addiction.

Although equating the mechanisms of tobacco addiction and those of host manipulation may appear obtuse, it is a clear way to demonstrate the gaps in the host manipulation research. Research on tobacco addiction may serve as a benchmark for the rigour needed in the search of mechanisms behind behavioural manipulation. Indeed, what is the causative *factor* that enables parasites to manipulate behaviour?

The jewel wasp-cockroach parasitoid-host system is one of the best understood examples of host behavioural manipulation. The wasp injects its venom directly into the cockroach’s brain (**Event A**). A component of the venom interferes with octopaminergic neurons (**Event B**), resulting in the loss of self-directed locomotion in the cockroach. This allows the wasp to lead the cockroach to its burrow [3, 13]. In both tobacco addiction and jewel wasp-cockroach system, nicotine and venom, respectively, unarguably connects **Event A** and **B**. Identification of the specific factors (nicotine and wasp’s venom) allows us to reliably conclude that the molecular changes observed in the smoker/host are a direct result of the cigarette/parasitoid and not just some other coincidental side effect of smoking/infection.

### Connecting parasite establishment and molecular changes

**Fig. 2:**
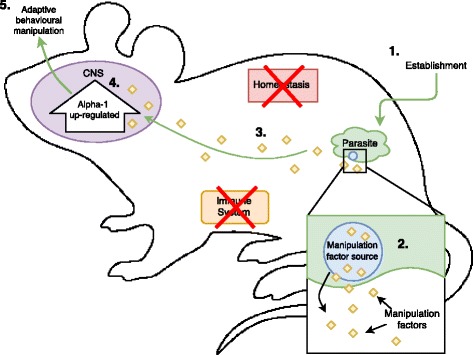
Hypothetical host-parasite system where the parasite is adaptively manipulating behaviour *via* Alpha-1 upregulation in the central nervous system (CNS) of the host. The major known (1, 4, 5) and hypothetical (2, 3) steps required for adaptive behavioural manipulation are presented here. Evidence of steps 2 and 3 has allowed researchers to eliminate the possibility that Alpha-1 upregulation was a side effect of infection. *Key*: Numbers represent the known and potential fundamental steps in host manipulation: (1) Establishment of parasite in host (location of parasite will vary depending of host-parasite system i.e. CNS, muscle, digestive tract); (2) Source (potentially multiple different sources) of manipulation factors activates at a given time during the parasite's development cycle, releasing manipulation factors into the host; (3) Manipulation factors exert their effects on one or more physiological systems; (4) Molecular change in the host (i.e. Alpha-1 upregulation as in the hypothetical case of Fig. 1) as a result on manipulation factors released by the parasite; (5) Host behavior changes as a result of the molecular change induced by the manipulation factors. Behavioural alteration directly increases the parasite's fitness. *Glossary*: *Manipulative factor*: Any molecule/substance released by the parasite that alters the normal functioning of one or more of the major identified pathways for host manipulation, resulting in a molecular shift in the host which ultimately changes the host behavior for the benefit of the parasite; *Manipulative factor source*: A structure (organelle, membrane, gland, enzyme etc.) which generates manipulative factors for the parasite to use in host manipulation

Drawing from the tobacco analogy and the jewel wasp, we can conceptualize a framework for how cases of adaptive behavioural manipulation should logically progress (Fig. 2). In order for parasites to directly manipulate their host’s behaviour, they should be releasing “manipulation factors” (see Fig. 2 for definition), similar in their functional role to nicotine or jewel wasp venom, which interact with the host’s physiological system(s). These “manipulation factors” should then result in the observed molecular/behavioural changes [7]. The source and production of manipulative factors (step 2) is also a key step that should not be overlooked either. Consider that in the jewel wasp and tobacco plant, the source of their manipulative factors, i.e. venom gland or roots respectively, is a key part of their manipulative process.

The three cases of host manipulation presented earlier are still missing evidence for manipulation factors and their source (step 2 and 3) compared to the tobacco addiction pathway and jewel wasp-cockroach parasite-host system (Table 1). The search for manipulation factors and their source in parasites suspected of adaptive behavioural manipulation should be a major goal of research in the area, as it directly connects parasite establishment with physiological change in the host. This bottom-up approach should be invaluable for finding the true molecular mechanisms involved in behavioural manipulation, as the impacts of the identified manipulative factor on host physiology can be logically tracked step-by-step. Comparatively, the alternative top-down approach of trying to elucidate the molecular changes in the host, before investigating step 2 and 3, makes it very difficult to validate the adaptive value of the observed molecular changes in the infected host. Again, in these top-down cases, it is entirely possible that the reported molecular changes are simply the host’s reaction (coping mechanism) to infection, rather than being enforced directly by the parasite (Fig. 1).

**Table 1:** Steps toward adaptive host manipulation compared against the known steps in *Toxoplasma*-rat, *Leishmania*-mouse, wasp-caterpillar and wasp-cockroach parasite-host systems. Additionally, tobacco addiction pathway included for comparison

Steps to adaptive host manipulation	Case 1	Case 2	Case 3	Jewel-wasp and cockroach	Tobacco addiction
**Step 1/Event A:** Parasite/tobacco establishment	*Toxoplasma gondii* infects a rat (*Rattus norvegicus*)	*Leishmania amazonensis* infects a mouse	Wasp *Cotesia congregata* injects its larvae into the catepilliar *Manduca sexta*	Wasp *Ampulex compressa* stings cockroach *Periplaneta americana*	Inhalation of tobacco smoke
**Step 2:** Manipulation factors released from source	?	?	?	Venom stored in the glands released into CNS	Nicotine released into lungs/brain from tobacco root
**Step 3:** Manipulation factors impacts physiological functioning	?	?	?	Neurotoxin in venom impacts octopaminergic neurons	Nicotine saturates acetylcholine receptors
**Step 4/Event B: **Molecular change in host	Testosterone release causes hypomethylation of the medial amygdala	Cytokine levels altered in pre-frontal cortex	Octopamine removal from hemolymph severely reduced	Sharp decrease in firing rate of affected neurons	Large dopamine release in the brain
**Step 5:** Behavioural change	Loss of innate aversion to cat odor	Set of anxiety behaviours	Suppression of feeding	Loss of self-directed movement	Addiction to tobacco

### Finding manipulation factors and their source

In order to validate this thought process, we need to have avenues, other than observation, for identifying the potential existence of manipulative factors and their source. Recently it was found in a stickleback-cestode host-parasite system that major changes in parasite gene expression occurred during the transition from the cestode’s intermediate host to its definitive host [14]. The transition from intermediate to definitive host is aided by loss of anti-predator behaviour in the intermediate host. Following the products of genes specifically switched on during transitional/manipulative phases in the parasite’s life-cycle could be an excellent place to start looking for manipulative factors. However, this may be an over-simplification. In this approach, we assume that the manipulative process in the parasite is a defined event initiated at a specific time, whereas achieving behavioural manipulation could instead result from a gradual process that starts early in the parasite’s life-cycle. Considering behavioural manipulation can range from a subtle change in pre-existing traits to the creation of entirely new behaviours, both punctual and gradual manipulation are distinct possibilities. Essentially, the aim here should be to pair gene expression changes in the parasite with behavioural changes in the host, and investigate these relationships for potential manipulative factors.

Finding the potential source of manipulation factors is equally important. Genomic analysis may allow researchers to identify simple manipulation factor sources such as enzymes. However, if the source is a complex structure (i.e. tissue, membrane, organelle) the number of genes involved in its development may make it difficult to identify it from genomic analysis alone, especially if that development is drawn out over the parasite’s life-cycle. Detailed histological analysis (or in-situ hybridization) of manipulative parasites, coinciding with adaptive behavioural changes in their host could serve to localise complex sources. The internal anatomy of parasites changes as they progress through their life-cycle (e.g. [15–18]). Therefore, it is not unlikely that a manipulation factor source may be absent early in a parasite’s life but present later on when manipulation occurs.

Host-parasite size disparity may also give insight into when the source becomes active. If host-parasite size disparity is great, we may expect a source that the parasite can store, as higher amounts of manipulation factor may be needed to impact host physiology. Alternatively, in host-parasite systems with little size disparity, such as hairworm-insect systems where the parasite induces its terrestrial host to seek and jump in water, the rate of production of manipulative factors may be high enough not to require storage. However, regardless of size disparity, sudden or rapid behavioural changes in the host may necessitate storage of manipulation factors by the parasite, whereas gradual behavioural changes in hosts may only require sustained production. Determining the probability that a parasite’s source of manipulation factor requires storage is important for two reasons: it informs histological searches for the source, and suggests when in the parasite’s life-cycle the source might appear.

Finally, it is also important to consider that parasites may repurpose or expand functionality of existing organelles/tissue for generating and secreting manipulation factors. Therefore, histological analysis should focus on existing structures/organelles as well as identification of new structures. Particular interest should be paid to existing organelles already capable of storage in high-disparity, rapid behavioural change host-parasite systems.

## Conclusion

Earlier studies often use the term ‘proximate mechanism’ when reporting physiological changes in infected hosts. Proximate is an incorrect epithet for these mechanisms. If adaptive behavioural manipulation is occurring in these cases, the physiological changes in the host are actually several steps downstream from the true cause; the manipulation factors themselves and their source are thus likely the true proximate mechanisms. Adopting this alternative perspective provides a bottom-up approach that has the potential to empirically differentiate an adaptive physiological manipulation from other possible consequences of infection. When implemented, this approach will likely accelerate progress in the field of behavioural manipulation.
